# The Encapsulation of Natural Organic Dyes on TiO_2_ for Photochromism Control

**DOI:** 10.3390/ijms24097860

**Published:** 2023-04-26

**Authors:** Hye Ju Lee, Jong Won Shim, Jung Jin Lee, Won Jun Lee

**Affiliations:** 1 Department of Fiber System Engineering, Dankook University, Yongin 16890, Republic of Korea; 2 Department of Applied Chemistry, Dongduk Women’s University, Seoul 02748, Republic of Korea

**Keywords:** titanium dioxide, photochromism, natural organic dye, encapsulation

## Abstract

Titanium dioxide (TiO_2_) plays a pivotal role in photocatalytic reactions and holds great promise for the cosmetic and paint industries due to its white color and high refractive index. However, the original color of TiO_2_ changes gradually to blue or yellow with UV irradiation, which affects its color realization. We encapsulated TiO_2_ with several natural organic dye compounds, including purpurin, curcumin, and safflower, to control its photochromism and realize a range of different colors. The chemical reaction between TiO_2_ and dyes based on their functional group was investigated, and the light absorption was tested via FTIR and UV–Vis spectroscopy. The changes in morphology and size distribution additionally supported their successful encapsulation. The discoloration after UV irradiation was evaluated by measuring the color difference (ΔE) of control TiO_2_ and dye encapsulated TiO_2_. The unique structure utilized natural dyes to preserve photochromism based on the physical barrier and automatically controlled the electronic transition of core TiO_2_. In particular, the color difference values of purpurin and curcumin were 4.05 and 3.76, which is lower than the 5.36 of the control TiO_2_. Dye encapsulated TiO_2_ was manipulated into lipstick to verify its color realization and retention.

## 1. Introduction

White titanium dioxide (TiO_2_), especially in the rutile phase, has been widely used as a photocatalytic ingredient due to its adequate band gap (i.e., 3.0 eV) [1,2,3], refractive index (i.e., 2.5) [4,5], and relatively low density (i.e., 3.78 g/cc). Indeed, their excellent control of particle sizes and facile dispersions in various solvents have powered their versatile use in real-life applications, such as pigments [6,7], paints [8,9], rubber [10], and cosmetics [11,12], for UV protection and permanent white color realization due to their outstanding chemical and physical stability. For color correction, TiO_2_ has certain advantages, including facile control of the particle size which efficiently scatters visible light and realizes a matte texture with a decrease in the particle size and clear opacity [13,14,15]. However, with their different particle sizes, TiO_2_ gradually changes from its original white color to yellow [16] or blue [17] (i.e., photochromism) which obscures the definition of permanent white. In principle, the oxidation state of Ti in TiO_2_ generally changes from Ti^4+^ to Ti^3+^ with a structural change in oxide on the surface of Ti under UV irradiation [18,19] which absorbs a shorter wavelength of light (i.e., blue shift) and displays a blue color.

To preserve the color degradation of TiO_2_, a common strategy is to control the chemical reduction of Ti with the incorporation of heterogeneous elements (1) on an atomic scale (i.e., doping) and (2) on a macroscopic scale (i.e., physical encapsulation). For example, with substitutional and interstitial atomic doping with nitrogen (N) [20,21,22,23], iron (Fe) [24], and nickel (Ni) [25,26], structural and electronic changes in Ti could be restricted in the presence of heterogeneous elements. Indeed, heterogeneous doping reduces the bandgap significantly which broadens the photocatalytic activation in the visible-light range [27]. However, heavy metals such as Fe and Ni could cause the color to change to red and result in the complex chemical reduction state of Ti which results in troublesome issues such as photodegradation and skin irritation [28]. For N-doping, nitrogen could lower the crystallinity of TiO_2_ and requires a high temperature (over 400 °C) and vacuum process. Physical encapsulation (i.e., heterogeneous incorporation on a macroscopic scale) has provided a shield on the surface of TiO_2_ which (1) reduces the amount of light and (2) increases the degree of light scattering. In this structure, the electronic configuration of Ti could be stabilized by the strong electrostatic attraction awarded from the shield material. Such physical encapsulation was found to guide color change as well as stable dispersion.

Natural organic dyes extracted from plants, fruits, and flowers can absorb visible light [29] and thereby display distinct colors together with efficient charge-transfer abilities. Considerable studies have been devoted to the hybridization of natural organic dyes with TiO_2_ to acquire (1) visible light photocatalysts [30,31] and (2) heterojunction solar cells [32,33]. In principle, the natural aromatic structure can be exploited to control the structure of the aggregate states of TiO_2_, enabling the photo-activated chemical reaction in the aforementioned applications. Along with strong visible absorption and charge transfer excitations, coloring permanent white TiO_2_ makes hybrid materials attractive ingredients for functional colored powders and pastes. The formation of a coordination bond between Ti^4+^ and the ligands of natural organic dyes physically awards the stable dispersion of hybrid particles without severe agglomeration, leading to the control of photochromism and outstanding color persistence. Indeed, diverse colors from numerous natural organic dyes could allow for the creation of warm tone or cool tone colored hybrid photocatalysts with genuine permanent white TiO_2_.

In this study, we propose an effective strategy that exploits the encapsulation of natural organic dyes on TiO_2_ particles and creates warm tone colored materials with (1) violet, (2) orange, and (3) yellow, ultimately preserving the photochromism. We demonstrate that introducing three different natural organic dyes, including (1) purpurin (1,2,4-Trihydroxyanthraquinone) [34], (2) curcumin (*Curcuma longa*), and (3) safflower (*Carthamus tinctorius*) [35], effectively mitigates photochromism and increases the dispersing ability of TiO_2_ in the fabrication process of color paste. The introduction of natural organic dyes increases the diameter of TiO_2_ hybrid particulates with increasing concentrations aided by their outstanding adsorption and penetration. The successful encapsulation of natural organic dyes on the surface of TiO_2_ through a coordination bond provides opportunities to control the photochromism with a warm tone color. CIE-L*a*b* investigation confirms color changes from white to (1) violet, (2) orange, and (3) yellow with different natural organic dye sources. Together, the manipulated hybrid structure contributes to the design of appropriate colored photocatalysts for use in powders and pastes for cosmetics.

## 2. Results and Discussion

### 2.1. Interaction between TiO_2_ and Natural Organic Dyes

**Figure 1:**
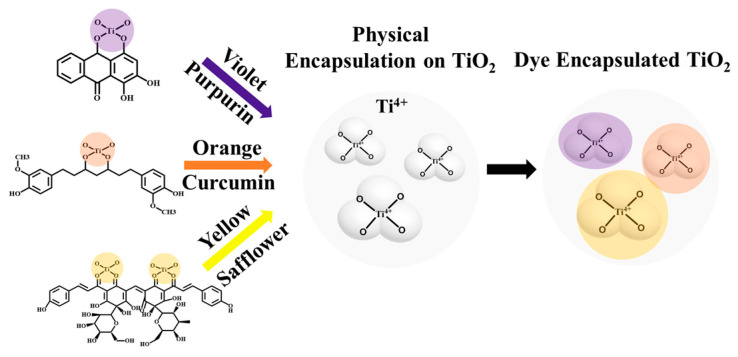
Schematic of dye encapsulation on TiO_2_ particulates.

The chemical encapsulation was conducted through the co-precipitation of TiO_2_ particles and natural organic dyes in an ammonia solution (c.a. 25~30 vol%) which results in the self-assembly of constituents with different colors (Figure 1). In brief, colored mixtures of TiO_2_ and dyes were initially centrifuged to remove lightweight and unreacted constituents, which was especially effective for residual dye removal. It should be noted that natural organic dyes are well dispersed in ammonia solutions [36]. Indeed, centrifugal separation was helpful for the separation of small TiO_2_ particles with certain density gradients [37]. After rinsing with DI water, the mixtures were collected on filter paper and dried at 80 °C without a vacuum. We hypothesized that the mixtures are chemically integrated with surface functional groups which boosts hybridization with physical depletion forces.

**Figure 2:**
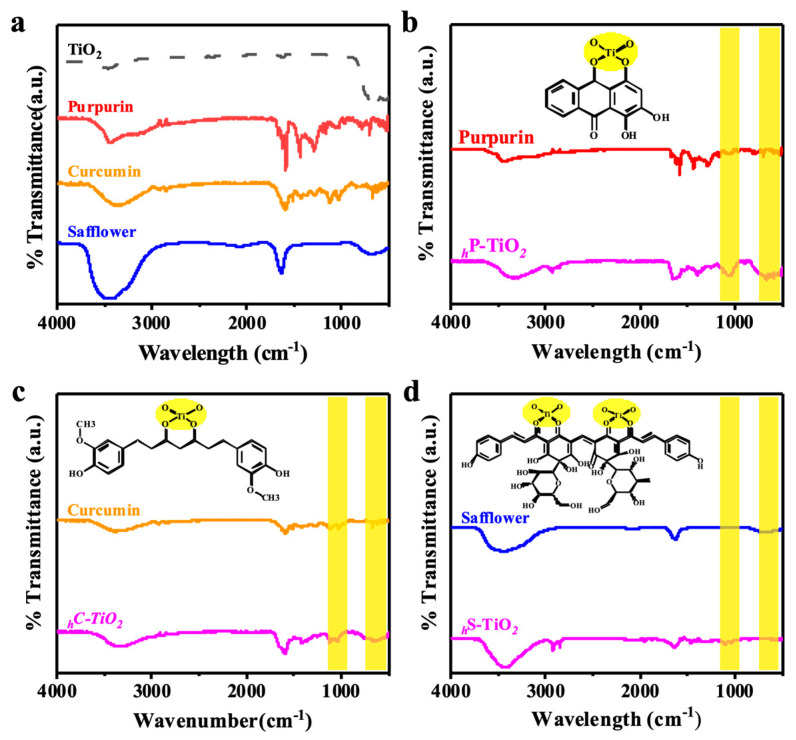
FTIR spectra of (**a**) control TiO_2_, (**b**) purpurin encapsulated TiO_2_, (**c**) curcumin encapsulated TiO_2_, and (**d**) safflower encapsulated TiO_2_.

To verify the potential interaction between TiO_2_ and natural organic dyes, the characteristic surface functional group was investigated with Fourier transform infrared spectroscopy (FTIR). Figure 2 shows the FTIR spectra of control TiO_2_, natural organic dyes, and their composites. For TiO_2_, two characteristic bands from O-H stretching and bending were observed near 3500 cm^−1^ and 1630 cm^−1^, respectively [38,39]. Note that these two peaks slightly shifted to higher frequencies after hybridization. Importantly, the natural organic dyes utilized here, including (1) anthraquinone purpurin, (2) turmeric curcumin, and (3) flavonoid safflower, each possess a conjugated system (i.e., a structure with a benzene ring). Therefore, strong stretching vibrations of the O-H bond (c.a. 3600–4000 cm^−1^), C-H bond (c.a. 400–1500 cm^−1^), and C=O (c.a. 1710–1720 cm^−1^) were observed for all dyes. Indeed, several minor peaks around 1350 cm^−1^ and 1000 cm^−1^ were found which could be attributed to the vibration of tertiary C-OH and the stretching of the =C-H group in the structure. Interestingly, there was a fluctuating broad band around 800 cm^−1^, potentially from Ti-O-C or Ti-O-Ti linkage, which could be altered by encapsulated dyes. However, the peak observed at 690 cm^−1^, corresponding to Ti-O bending, was significantly shifted to 614 cm^−1^, which indicates that the Ti-O bond was weakened by the encapsulation of dyes on the surface. Note that the peak shift was commonly observed at a high concentration (i.e., 1 wt%) of all dyes on the TiO_2_ surface. For safflower, the shift was less evident due to the complex conjugation of its aromatic structure (Figure 2d). The experimental results of FTIR spectra indicate that dyes could be progressively encapsulated on TiO_2_ with Ti-O-C linkage. The carbonyl functional group in various dyes plays a pivotal role in chemical interactions.

**Figure 3:**
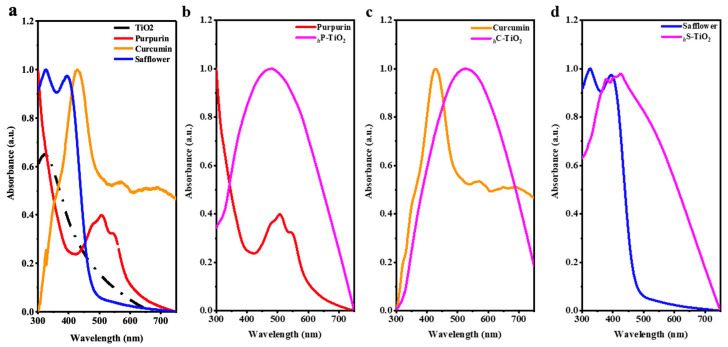
UV–Vis spectra of (**a**) control TiO_2_, (**b**) purpurin encapsulated TiO_2_, (**c**) curcumin encapsulated TiO_2_, and (**d**) safflower encapsulated TiO_2_ with a low concentration of 1.0 wt%.

Ultraviolet–visible (UV–Vis) spectroscopy was utilized to confirm the light absorption and optical properties of the control TiO_2_, the natural dyes (i.e., purpurin, curcumin, and safflower), and their composites (Figure 3). For clarity, here, we focus only on the absorption peaks from the constituents, but the enhancement can be translated to the specific band diagram model in further research. Control TiO_2_ showed a typical UV–Vis absorption spectrum of rutile TiO_2_ particles at 320 nm. (Figure 3a) which confirms that TiO_2_ reacts in UV rays, mainly with its electron transition from Ti^4+^ to Ti^3+^. For purpurin, the spectra showed three main peaks in the visible light region at 480 nm, 520 nm, and 560 nm when it was dispersed in water. Note that the peaks could be different in different solvents of DMSO with solute–solvent interactions. Interestingly, the encapsulation of TiO_2_ with purpurin showed moderate absorption in the UV-B region but a dramatic increase in absorption in the visible light region which is attributed to the introduction of purpurin on the surface (Figure 3b). The characteristic absorption peak from purpurin were similarly observed in the purpurin encapsulated TiO_2_ around 500 nm due to the the π-π* transition. Notably, the enhancement in visible light became clearer between 400 nm and 600 nm due to the existence of purpurin, which provides evidence of a favorable reaction. The peak wavelength shift from 320 nm to 480 nm was mainly attributed to the existence of rutile TiO_2_ as observed in control sample (Figure 3a). Note that the absorption in the UV-B region was maintained with the introduction of purpurin. Similarly, the absorption in the UV-B region was affected by the encapsulation of curcumin (Figure 3c). Curcumin showed a characteristic absorption peak at 427 nm and a band between 450 nm and 550 nm. Note that the enol functional group in curcumin was the main cause of the transition at 427 nm. Since the surface of the TiO_2_ was mostly covered with curcumin, the absorption in the UV-B region was negligible. However, the introduction of curcumin changed the characteristic absorption band between 500 nm and 550 nm, which is comparable to the purpurin encapsulated TiO_2_. This may be attributed to the amount of curcumin as observed in FTIR spectra. In the case of safflower encapsulation, a significant absorption enhancement in the UV-B region was observed together with a slight increase in the visible light region. Safflower exhibited characteristic absorption peaks at 310 nm and 425 nm. Due to a weak transition in the visible light region itself, the safflower encapsulated TiO_2_ showed limited transition in visible light region. A slight transition between 400 nm and 425 nm may be attributed to the limited surface coverage of safflower. Safflower has a larger molecular weight (i.e., 910.8 g/mol) which has a high radius of gyration. Therefore, the large molecule of safflower could cause steric hindrance on the surface of TiO_2_, causing partial encapsulation in the chemical reaction. Most importantly, control rutile TiO_2_ exhibited a transition only in the UV-B region, which could be shifted to the visible light region with the introduction of dyes. Meanwhile, the linkage between TiO_2_ and dyes with Ti-O-C linkage may ultimately affect the band diagram which shows a distinct absorption band in the visible light region between 450 nm and 600 nm. The UV–Vis spectra provide their degree of encapsulation qualitatively, which is supported by the morphology and CIE L*a*b* color analysis discussed in the next section.

**Figure 4:**
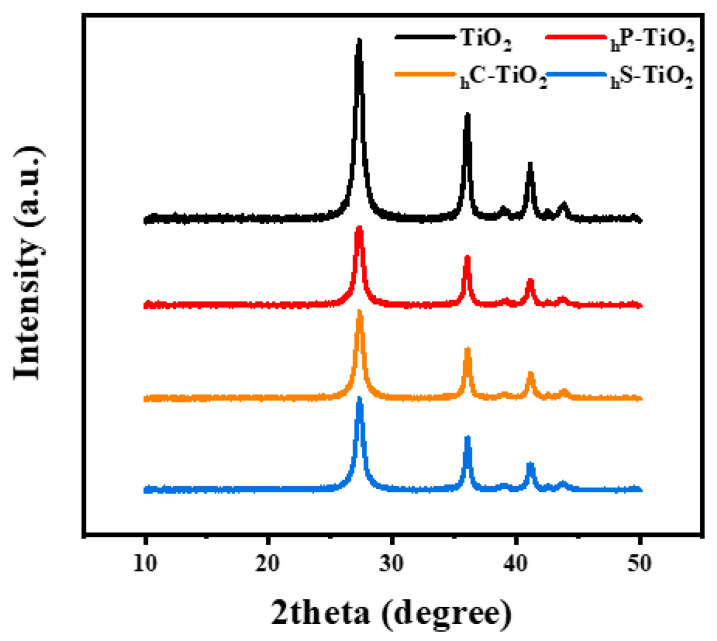
XRD diffractograms of TiO_2_ (black, top), purpurin encapsulated TiO_2_ (red), curcumin encapsulated TiO_2_ (orange), and safflower encapsulated TiO_2_ (blue, bottom).

To identify the phase of TiO_2_, X-ray diffraction (XRD) was implemented. As shown in Figure 4, control TiO_2_ nanoparticles had crystalline properties and displayed characteristic diffraction peaks at 27.3° (110), 36.1° (101), and 41.2° (111), which are in good agreement with the tetragonal rutile phase (SG, P42/mmm; JCPDS no. 88–1175, a = b = 0.4517 nm and c = 0.2940 nm) [40]. Interestingly, the dye encapsulated samples together with the curcumin and safflower showed similar XRD profiles as the control TiO_2_. While curcumin was found to show a diffraction pattern [41], the low concentrations of curcumin (e.g., 1.0 wt%) were not enough to present a diffraction pattern from the curcumin encapsulated TiO_2_. However, the preserved rutile phase after dye encapsulation indicates that the presence of dyes has a low impact on the ordered arrangement of TiO_2_. Note that rutile is the most thermodynamically stable phase for TiO_2_ [42].

### 2.2. Size Distribution after Dye Encapsulation on TiO_2_

**Figure 5:**
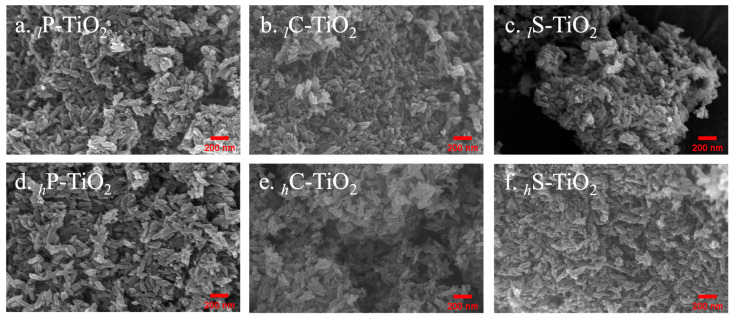
HR-SEM images of TiO_2_ natural organic dye nanocomposites. (**a**) Purpurin encapsulated TiO_2_ (0.1 wt%), (**b**) curcumin encapsulated TiO_2_ (0.1 wt%), (**c**) safflower encapsulated TiO_2_ (0.1 wt%), (**d**) purpurin encapsulated TiO_2_ (1.0 wt%), (**e**) curcumin encapsulated TiO_2_ (1.0 wt%), and (**f**) safflower encapsulated TiO_2_ (1.0 wt%).

Based on the spectroscopic results, we confirmed the successful encapsulation of natural organic dyes on TiO_2_ surfaces, which provides certain information about their favorable reaction. The encapsulation effect can be found in their size, particularly the agglomerate size of particles. Analyses using scanning electron microscopy (SEM) were performed to calculate the size of agglomerates after spin coating on SiO_x_ substrates (1 × 1 cm^2^). Interestingly, the size of the TiO_2_ increased dramatically after encapsulation (Figure S1). In particular, we focused on their particle sizes and their histograms because the phase separation between the dyes and TiO_2_ could lead to a bimodal distribution if there is no chemical interaction. Overall, the encapsulated TiO_2_ particles showed a continuous increase in particle size in SEM without any phase separation. The SEM images, presented in Figure 5, show that the diameter of TiO_2_ with concentrations of 0.1 wt% and 1.0 wt% of (a) purpurin, (b) curcumin, and (c) safflower increased without poor agglomeration or phase separation. Considering the shape of the needle-like TiO_2_, the particle sizes were measured based on length for the sake of clarity. Note that the particle length provided in the sample specifications was 80 nm. Briefly, the length of 94.8.7 ± 3.7 nm for the TiO_2_ was increased to 116.5 ± 3.4, 120.2 ± 3.3, and 113.3 ± 3.4 nm with a 1 wt% introduction of purpurin, curcumin, and safflower, respectively, which is summarized in Table 1. Importantly, the particle size was correlated with the degree of encapsulation expected from UV–Vis spectra. As noted, while safflower has a chemical affinity to TiO_2_, the steric hindrance due to the high molecular weight could interfere with the adhesion at an atomic level, resulting in the smallest length with high deviation. Indeed, because curcumin has full coverage with an excellent chemical affinity, the length of curcumin encapsulated TiO_2_ achieved the largest length (i.e., 120.2 ± 3.3 nm). The different agglomeration behavior in SEM may also be attributed to their chemical affinity and degree of encapsulation as previously observed in FTIR and UV–Vis spectra. Next, we investigated the effects of a series of different concentrations of dyes on TiO_2_ surfaces from a low concentration of 0.1 wt% to a high concentration of 1.0 wt%. Note that encapsulation over 1 wt% of dye was excluded due to the significant residual dyes after the centrifugation process in the experimental stage. The average particle size, summarized in Table 1, shows that encapsulation proceeded linearly with an increasing concentration of dyes that reached values up to 112.1 ± 3.9 nm for 0.1 wt% (i.e., *_l_*C-TiO_2_) and 120.2 ± 3.3 nm for 1.0 wt% (i.e., *_h_*C-TiO_2_) with curcumin. Interestingly, while safflower has a lower chemical affinity, the thickness or particle size increased as the concentration of dyes increased, suggesting that coverage could be enhanced with a certain level of concentration.

### 2.3. Color Features of TiO_2_ after Dye Encapsulation

**Figure 6:**
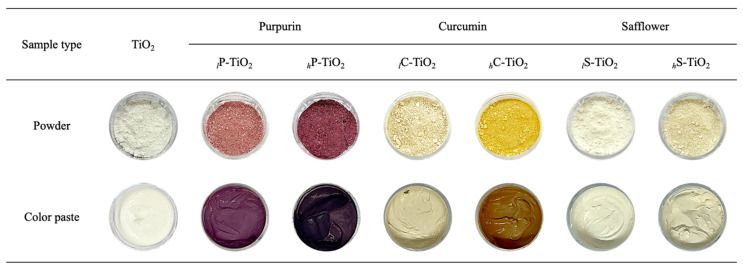
Digital images of powder and color paste with TiO_2_ natural organic dye nanocomposites.

To elucidate the color changes induced by the encapsulation of natural organic dyes, we recorded digital photographs for all powders and colored pastes (i.e., powder dispersed in DI water, green gum, castor oil, and carnauba wax; see details in the experimental procedure). Note that the digital photographs were taken in the same conditions (Figure 6). The digital photographs for all of the powder samples confirmed the color change from white to violet, orange, and yellow with purpurin, curcumin, and safflower, respectively.

With an increased concentration, the color of powder became darker due to the color saturation, which was explored in the CIE-L*a*b* analysis. As shown at the bottom of Figure 5, we checked whether the encapsulated dye detached in the preparation of color pastes. However, none of the encapsulated TiO_2_ showed phase separation or significant agglomeration in the process. To identify the color in detail, CIE-L*a*b* analysis was conducted. Considering the direct application of TiO_2_ for color cosmetics, CIE-L*a*b* was chosen to depict the approximate color for human vision. In CIE-L*a*b*, L* refers to perceptual lightness, a* refers to red and green colors of human vision, and b* refers to blue and yellow colors of human vision. For L*, the lightness value is 0 for black and 100 for white. The a* axis represents the green–red opponents with negative numbers for green and positive numbers for red. Meanwhile, the b* axis represents the blue–yellow opponents with negative numbers for blue and positive numbers for yellow.

Table 2 shows the representative L*, a*, and b* values obtained from all of the different samples in paste form. Notably, we found that the value of L* decreased with encapsulation from 113.3 to 43.8, 77.5, and 110.1 with purpurin, curcumin, and safflower, respectively, suggesting a progressive reduction in perceptual lightness. This is attributed to the coverage effect of dyes on the TiO_2_ surface, which was observed in UV-Vis spectra. As observed in Figure 6, the original white color turned into darker violet, orange, and yellow with increasing concentrations which are reflected in L* values. Interestingly, for purpurin, a small amount of encapsulation reduced the L* value dramatically, which is mainly due to the strong color saturation. As observed in the digital photographs, with the encapsulation of purpurin, the color changed to a dark violet color consistently with increasing concentrations. Note that there was no linear relationship between the L* value and the degree of encapsulation predicted above. However, consistent with the original a* and b* values from curcumin [43], the color paste with *_h_*C-TiO_2_ showed positive values of 17.9 and 31.2 for a* and b*, respectively, which was the most dramatic shift in values compared with the other samples with purpurin and safflower. Similar to the UV-Vis spectra, the curcumin encapsulation was found to consistently be the most effective for color correction. In contrast, color pastes with S-TiO_2_ showed lower shifts in a* and b* values, which reflects their limit of encapsulation on TiO_2_. These changes in L*, a*, and b* with the encapsulation are commensurate for photochromism control, which is observed with UV irradiation.

### 2.4. Preserving Photochromism with Dye Encapsulation

**Figure 7:**
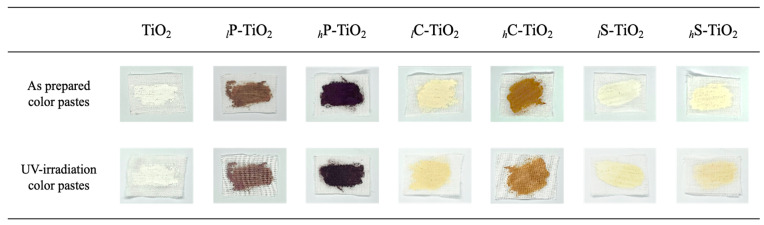
Photochromism of color paste with TiO_2_ with different dye encapsulations.

We observed that encapsulation changed their original color effectively in human vision, turning light pale blue–white to a warm tone color with small amounts of dyes, i.e., less than 0.1 wt%. Indeed, the dye encapsulation exhibited comparably beneficial effects, enhancing the stability of TiO_2_ under UV irradiation. To observe the color change after UV irradiation (i.e., photochromism), digital photographs were taken before and after UV exposure for 3 days (Figure 7). Note that all measurements were investigated on slurry-cast white cotton pads with exactly the same amounts.

**Figure 8:**
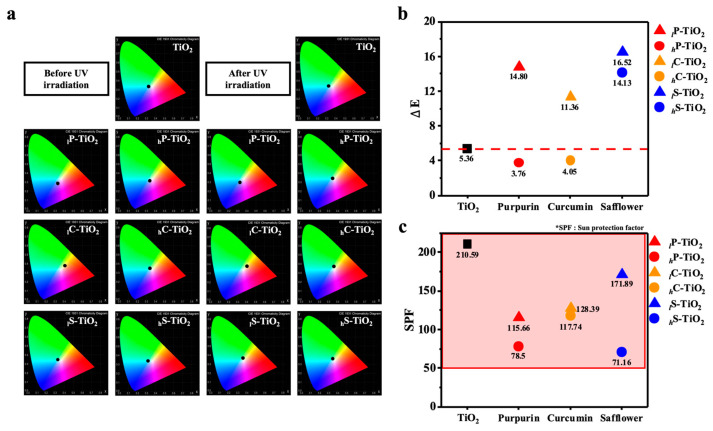
Evaluation of photochromism. (**a**) CIE L*a*b* 1931 chromaticity diagram, (**b**) color difference (∆E), and (**c**) the sun protection factor of TiO_2_ natural organic dye nanocomposites. CIE-L*a*b* was chosen to depict the approximate color for human vision. In CIE-L*a*b*, L* refers to perceptual lightness, a* refers to red and green colors of human vision, and b* refers to blue and yellow colors of human vision. Black dot in the center of chromaticity diagram using a black background provides a framework for specification of color sensation. In (**b**,**c**) square, triangle, and circle represents the control sample (bare TiO_2_), 0.1 wt% dye encap-sulated TiO_2_, and 1.0 wt% dye encapsulated TiO_2_, respectively. Red, green, and blue represents purpurin, curcumin, and safflower, respectively.

Interestingly, we observed that the color pastes with dye encapsulated TiO_2_ underwent a relatively dramatic change in appearance, turning from the original light violet, orange, and yellow to pale colors with a change in texture. Note that the change in texture may be attributed to the drying or degradation effect after 3 days of UV irradiation. However, human vision or digital photographs are not adequate for the judgment of color retention. To determine the degree of discoloration, CIE-L*a*b* values were compared before and after UV irradiation with a chromaticity diagram. Since the L*a*b* model has three axes, a three-dimensional space could be depicted in the space to display the full gamut of colors in detail (Figure 8). Since UV irradiation affects the electronic structure of TiO_2_ and dyes, the effect is clearly visible in the chromaticity diagram. For safflower with 1.0 wt% encapsulation, the black dot at the center of the chromaticity diagram was shifted toward green after UV irradiation. However, for curcumin with 1.0 wt% encapsulation, the black dot maintained the center position with less shifting. The color difference value (ΔE) was precisely estimated to compare the degree of shift after UV irradiation using Equation (1).

(1) $$\Delta E=\sqrt{{\left(L^{*}{}_{\mathrm{before}}-L^{*}{}_{\mathrm{after}}\right)}^{2}+{\left(a^{*}{}_{\mathrm{before}}-a^{*}{}_{\mathrm{after}}\right)}^{2}+{\left(b^{*}{}_{\mathrm{before}}-b^{*}{}_{\mathrm{after}}\right)}^{2}}$$

**Figure 9:**
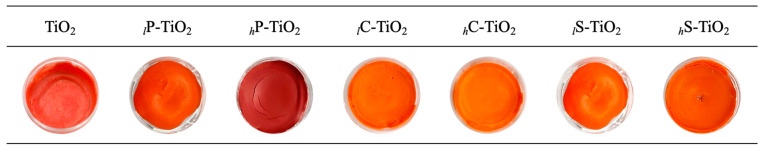
Digital photographs of lipstick with dye encapsulated TiO_2_.

By calculating the color difference value (ΔE), which represents the degree of discoloration, we quantified the effect for preserving photochromism after UV irradiation. Surprisingly, we noticed that the control TiO_2_-based color paste also showed a significant amount of discoloration (ΔE = 5.36) over 3 days of UV irradiation, which differed slightly from our judgment using human vision and photographs. In contrast, and most importantly, with 1 wt% of purpurin and curcumin, the dye encapsulation improved this aspect, showing lower degrees of discoloration (ΔE) of 4.05 and 3.76, respectively. Note that 0.1 wt% of dye encapsulation provides a higher level of discoloration of 14.80 and 11.36, which may be attributed to insufficient encapsulation to interfere with the absorption of UV light in accordance with previous results. Indeed, at low dye concentrations, the lightfastness could be limited with accelerated degradation of chromophores [44]. The sun protection factor (SPF) was additionally estimated to quantify how much surface area of TiO_2_ was covered with the encapsulation. Originally, the SPF value stands for what percentage of material could protect against UV rays. We hypothesized that an increase in coverage may decrease the SPF factor, i.e., a reduction in the TiO_2_ surface area should decrease the SPF factor. The SPF value of the control TiO_2_-based color pastes was 210.6. When TiO_2_ was entirely encapsulated with curcumin, the SPF value was found to be 117.7, which was almost half of the control. The lower SPF supported that more effective encapsulation underwent the preparation process. It is noteworthy that the encapsulation of safflower did not effectively control the color and photochromism while maintaining the SPF factor. Lastly, we fabricated the red colored pastes with dye encapsulated TiO_2_ to verify the warm tone color realization (Figure 9 and Figure S2). Conventional Eosin Y was chosen for realizing a red color. As observed, the incorporation of dye encapsulated TiO_2_ affected the color with different dyes and concentrations. It should be highlighted that the introduction of dye encapsulated TiO_2_ finely altered the color of lipstick with a moderate SPF factor, together with very little discoloration.

## 3. Materials and Methods

### 3.1. Materials

TiO_2_ powder (TELIKA_NC, width = 20 nm, length = 80 nm, Rutile, TAEKYUNG SBC, Gunsan, Republic of Korea) was used as received without further purification. Three natural organic dyes, including purpurin, curcumin, and safflower, were used as received without further purification. Purpurin (CAS no. 81-54-9) and curcumin (CAS no.458-37-7) powders, Eosin Y solution (CAS no.17372-87-1), and deionized water (ACS reagent, 320072) were purchased from Sigma Aldrich, St. Louis, MO, United States. Safflower extract was supplied by Chung Do Chemical Corporation Co., Ltd., Gunsan, Republic of Korea. Ammonia solution (extra pure, 25.0~30%) was supplied by Samchun Chemical, Pyeongtaek, South Korea. Conventional copernicia prunifera (Carnauba) wax (CAS no. 8015-86-9), castor oil (CAS no. 8001-79-4), and polysaccharides (CAS no. 71010-52-1) were purchased from a local drug store for the fabrication of color paste.

### 3.2. Self-Encapsulation of Natural Organic Dyes on TiO_2_

The self-encapsulation process was proceeded by the following steps. (1) TiO_2_ powder (10 g) was dispersed in an ammonia solution (25~30%, 100 mL) with mild stirring (300 rpm) at RT for an hour to induce Ti-N complex on the surface of TiO_2_. (2) Natural organic dyes were introduced at two different concentrations—(1) 0.1 mg/mL and (2) 1 mg/mL—to the prepared TiO_2_–ammonia solution with mild stirring (300 rpm) at RT for an hour. (3) Residual extraction was completed to remove extra natural organic dyes with the centrifugation process at 5000 rpm at 50 °C for 30 min. (4) Water and ammonia were eliminated with heat treatment at 80 °C in air conditioning for 2 h. All the collected samples were soaked and rinsed with DI water which was repeated at least two times.

### 3.3. Preparation of Color Pastes

Color paste was prepared in the following manner. (1) Solid extracts from anionic polysaccharides (i.e., gellan gum, 120 mg) were introduced into DI water (4 g, 1 g/cc) with mild stirring (300 rpm) at RT for 30 min. (2) Carnauba wax (120 mg) was introduced into castor oil (10 g, 0.95 g/cc). (3) Polysaccharide aqueous solution (step 1) and carnauba wax/castor oil mixtures (step 2) were blended with mild stirring (300 rpm) at 80 °C for 30 min. (4) The control (pure TiO_2_) and prepared colored TiO_2_ hybrid particles (2.5 g) were introduced into the above paste (14.24 g of gum/wax/oil mixture) to a set 15 wt% concentration with mild stirring (300 rpm) at 80 °C for 30 min and cooled down to RT. (5) To eliminate the air bubbles inside the mixture, all viscous pastes were rotated in a planetary centrifugal mixer (ARE-310, THINKY, CA, United States) at 2000 rpm for 30 min at RT. Color paste for lip care was also prepared together with the addition of Eosin Y solution after the above process.

### 3.4. Preparation of the Red Colored Pastes

Color paste for lip care (i.e., lipstick) was additionally prepared together with the introduction of Eosin Y solution. Lipstick was prepared in the following manner: (1) carnauba wax (80 mg) and TiO2 hybrid particulates (20 mg) were blended together with a mechanical milling process; (2) Eosin Y solution (60 mg, 1.015 g/cc) was introduced into castor oil (2.4 g, 0.95 g/cc); and (3) the mixture (step 1) and Eosin Y solution/castor oil mixtures (step 2) were blended with mild stirring (300 rpm) at RT for 30 min.

### 3.5. Characterization of TiO_2_ Hybrid Particulates

The colored TiO_2_ hybrid particulates were characterized with Fourier transform infrared spectroscopy (FTIR), high resolution scanning electron microscopy (HR-SEM), X-ray diffractometer (XRD), and ultraviolet–visible (UV–Vis) spectrophotometry. UV–Vis (Perkin Elmer Lambda 25, MA, United States) was used to determine the reflectivity properties from the absorption spectra of TiO_2_ hybrid particulates. UV–Vis measurements were carried out in a quartz cuvette ell at room temperature (25 °C) with a spectral range from 190 nm to 1100 nm. The distance between the lamp and sample was set to 121 mm with double beam light sources (pre-aligned Deuterium and Tungsten) and a linear absorbance up to 3.2 A. Samples for FTIR spectroscopy were prepared on a quartz plate. FTIR spectra were obtained using spectrum two (Perkin Elmer Spectrum Two, MA, United States) in an attenuated total reflection mode with a resolution of 4 cm^–1^. HR-SEM (Hitachi S-5200, Tokyo, Japan) was used to characterize the changes in particle sizes and dispersions of TiO_2_ after encapsulation. XRD (Bruker D8, MA, United States) measurements were performed inside a vacuum chamber to avoid scattering by air. Measurements were carried out using Cu-Kα radiation (λ = 1.5418 Å) for the 2θ range of 10° to 50° with a step size of 0.02°.

### 3.6. Characterization of Color Paste

The color pastes with TiO_2_ natural organic dye nanocomposites were characterized with a colorimeter (CIE L*a*b*) and sun protection factor analyzer system. CIE L*a*b* (X-rite, MI, United States) was used to characterize the optical properties of TiO_2_ hybrid particulate after ultraviolet irradiation. SPF (Solar Light Company, PA, United States) was used to investigate the sun protection effect of TiO_2_ after dye encapsulation.

## 4. Conclusions

In conclusion, we have demonstrated an effective molecular strategy based on dye encapsulation for preserving the photochromism of TiO_2_ which enables the realization of perceptual color and color retention in cosmetic applications. To promote facile adhesion between TiO_2_ and natural dyes during encapsulation, we investigated several functional groups of organic dyes. The degree of light absorption under UV–-Vis radiation provided clues regarding the effectiveness of encapsulation with a certain amount of coverage. In the core–sheath structure, the overall particulate size linearly increased with increasing concentrations of dyes without phase separation or agglomeration. We systematically investigated discoloration and brightness with UV irradiation. We assume that the existing natural dye compounds on the TiO_2_ surface contribute to preserving photochromism through two mechanisms: (1) the physical placement of the sheath layer (i.e., dye) which may reflect or absorb UV rays approaching the core TiO_2_ and (2) the electronic transition of Ti which may be effectively restricted by less absorbed energy. The color difference value (ΔE) from the curcumin encapsulated TiO_2_ confirmed that photochromism control was successfully achieved compared with the bare TiO_2_. Although the SPF factor was decreased, encapsulation with dye was also effective for color realization and color retention as observed through the fabrication of cosmetic color pastes (i.e., lipstick). Our encapsulation process is simple and straightforward for manufacturing cosmetic ingredients, and this work provides an important and practical design strategy to manipulate color features resulting from light reflection, transmission, and absorption.

## Figures and Tables

**Table 1:** Size distributions of control TiO_2_ and dye encapsulated TiO_2._

Sample Name		Particle Sizes (nm)
TiO_2_		94.8 ± 3.7
Purpurin	*_l_*P-TiO_2_	111.8 ± 2.6
	*_h_*P-TiO_2_	116.5 ± 3.4
Curcumin	*_l_*C-TiO_2_	112.1 ± 3.9
	*_h_*C-TiO_2_	120.2 ± 3.3
Safflower	*_l_*S-TiO_2_	88.3 ± 3.1
	*_h_*S-TiO_2_	113.3 ± 3.4

**Table 2:** CIE-L*a*b* analysis of color pastes based on different dye encapsulations.

Sample Name		L*	a*	b*
Control	TiO_2_	100	−1.3	5.2
Purpurin	*_l_*P-TiO_2_	78.2	13.5	−1.8
	*_h_*P-TiO_2_	43.8	14.4	−6.9
Curcumin	*_l_*C-TiO_2_	100	−0.5	14.3
	*_h_*C-TiO_2_	77.5	17.9	31.2
Safflower	*_l_*S-TiO_2_	100	−2.0	5.3
	*_h_*S-TiO_2_	100	−2.4	10.3

## Data Availability

The data presented in this study are available upon reasonable request.

## Supplementary Materials

The following supporting information can be downloaded at: https://www.mdpi.com/article/10.3390/ijms24097860/s1.
