# Supplementary material for: The Encapsulation of Natural Organic Dyes on TiO2 for Photochromism Control
Source: Int J Mol Sci. 2023 Apr 26;24(9):7860. doi: 10.3390/ijms24097860 (PMC10178252; doi:10.3390/ijms24097860)
Supplement: Supplementary file 1 [file ijms-24-07860-s001.zip › ijms-2277911-supplementary.pdf]

Supplementary Materials

The encapsulation of natural organic dyes on TiO_2 for photochro mism control

Hye Ju lee [1], Jong Won Shim [2], Jung Jin Lee[1], Won Jun Lee[1,*]

[1]Department of Fiber System Engineering, Dankook University, Yongin 16890, Republic of Korea
[2]Department of Applied Chemistry, Dongduk Women's University, Seoul, 02748 Republic of Korea

Contents

Figure S1: A representative SEM image of control TiO_2 used in this study\

Figure S2: (a) CIE L*a*b* 1931 chromaticity diagram, and (b) tabulated value of L*, a*, and b* from red colored pastes

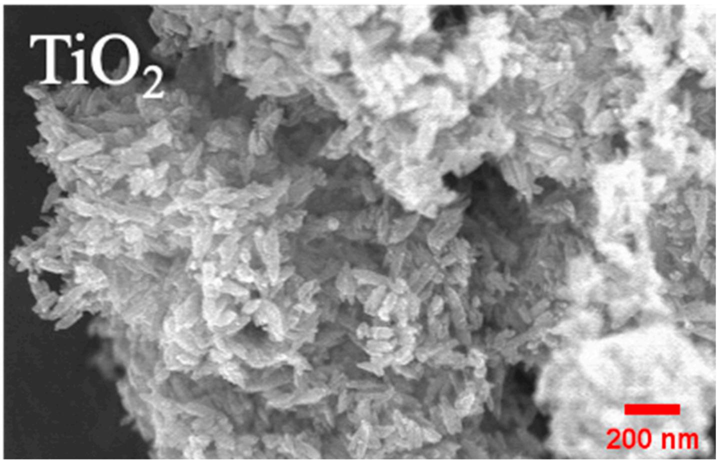

Figure S1. A representative HR-SEM image of control TiO_2 used in this study

a

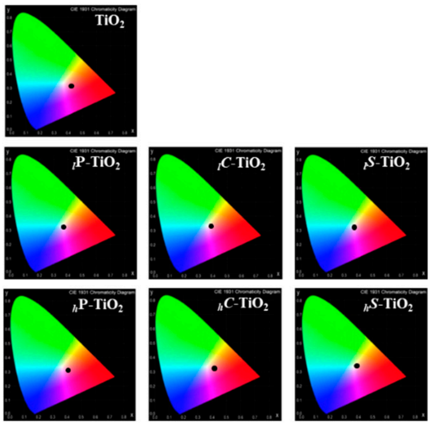

b

Sample name		L*	a*	b*
Control	TiO_2	80.528	49.305	13.914
Purpurin	$_l$P-TiO_2	88.125	28.521	18.631
	$_h$P-TiO_2	88.306	27.752	8.838
Curcumin	$_l$C-TiO_2	86.511	29.873	15.076
	$_h$C-TiO_2	83.443	43.188	17.409
Safflower	$_l$S-TiO_2	71.272	41.678	9.375
	$_h$S-TiO_2	86.843	26.164	7.678

Figure S2. (a) CIE L*a*b* 1931 chromaticity diagram, and (b) tabulated value of L*, a*, and b* from red colored pastes
